# Examining primary care physician rationale for not following geriatric choosing wisely recommendations

**DOI:** 10.1186/s12875-021-01440-w

**Published:** 2021-05-15

**Authors:** Theresa A. Rowe, Tiffany Brown, Jason N. Doctor, Jeffrey A. Linder, Stephen D. Persell

**Affiliations:** 1 Division of General Internal Medicine and Geriatrics, Department of Medicine, Northwestern University Feinberg School of Medicine, 750 N Lakeshore Dr. 10th Floor, Chicago, IL 60611 USA; 2 Sol Price School of Public Policy, University of Southern California, Los Angeles, CA USA; 3 Center for Primary Care Innovation, Institute for Public Health and Medicine, Feinberg School of Medicine, Northwestern University, Chicago, IL USA

**Keywords:** Choosing Wisely, Overuse, Older adults

## Abstract

**Background:**

The objective is to understand why physicians order tests or treatments in older adults contrary to published recommendations.

**Methods:**

Participants: Physicians above the median for ≥ 1 measures of overuse representing 3 Choosing Wisely topics. Measurements: Participants evaluated decisions in a semi-structured interview regarding: 1) Screening men aged ≥ 76 with prostate specific antigen 2) Ordering urine studies in women ≥ 65 without symptoms 3) Overtreating adults aged ≥ 75 with insulin or oral hypoglycemic medications. Two investigators independently coded transcripts using qualitative analysis.

**Results:**

Nineteen interviews were conducted across the three topics resulting in four themes. First, physicians were aware and knowledgeable of guidelines. Second, perceived patient preference towards overuse influenced physician action even when physicians felt strongly that testing was not indicated. Third, physicians overestimated benefits of a test and underemphasized potential harms. Fourth, physicians were resistant to change when patients appeared to be doing well.

**Conclusions:**

Though physicians expressed awareness to avoid overuse, deference to patient preferences and the tendency to distort the chance of benefit over harm influenced decisions to order testing. Approaches for decreasing unnecessary testing must account for perceived patient preferences, make the potential harms of overtesting salient, and address clinical inertia among patients who appear to be doing well.

**Supplementary Information:**

The online version contains supplementary material available at 10.1186/s12875-021-01440-w.

Practice guidelines often recommend different criteria for treatment or testing in older adults [1–3]. Specifically, The Choosing Wisely Campaign, an initiative of the American Board of Internal Medicine Foundation, endorsed by the American Geriatric Society, developed recommendations to help reduce potentially harmful tests and treatments [4]. Three recommendations relevant to primary care include: do not recommend prostate cancer screening for men over 75 years of age without considering life expectancy and the risks of testing, overdiagnosis, and overtreatment; do not use antimicrobials for bacteriuria in older adults unless specific urinary tract symptoms are present; and avoid medications other than metformin to achieve hemoglobin A1C (HbA1C) < 7.5% in most older adults. However, many older adults still receive these tests and treatments [5–8]. Recently, we found over utilization in these three areas to be quite common ranging from 23% for overuse of prostate specific antigen (PSA) screening and urine testing to 30% for overuse of insulin or oral hypoglycemics [8]. Variation among clinicians was substantial in ordering the examined services [8].

Several studies have explored why some clinicians over-utilize tests and treatments of unlikely benefit in older adults [9, 10]. Lack of guideline knowledge, practice inertia, patient preference, and underestimation of risks associated with tests and treatments have been identified as barriers to guideline adherence [10, 11]. However, improvement interventions have not specifically targeted clinicians who most frequently overuse. To our knowledge, this is the first study to do a focused qualitative examination of physicians who over-order tests and treatments contrary to Choosing Wisely recommendations.

Understanding the perceptions of clinicians who most frequently over-order tests and treatments contrary to published recommendations could inform interventions to change these behaviors. Therefore, we utilized qualitative methods to explore perspectives and barriers to guideline adherence from clinicians who are high over-users in the three areas mentioned above.

## Methods

### Design and setting

This was a qualitative study using Constant Comparative Thematic Analysis. We conducted semi-structured interviews lasting 20–30 min with high-utilizing primary care physicians caring for older adults from primary care clinics within one large health system in Chicago, IL from 2017–2018. Northwestern University’s institutional review board approved the study.

### Participants

We recruited physicians who were above the median on at least one of the following measures: 1) PSA screening in men aged ≥ 76 years, 2) urinalysis or urine culture testing in women aged ≥ 65 years without specific genitourinary or infectious signs or symptoms, and 3) oral hypoglycemic medication or insulin prescription in adults aged ≥ 75 years with diabetes and a HbA1C < 7.0%. All physicians above the median on at least one topic were contacted and recruited through direct email. Quality measure performance was extracted from electronic health record data. Details and specifications for these quality measures have been published [8]. We excluded physicians with fewer than 10 eligible patients. Each participant was interviewed on 1 to 3 of the overtreatment topics. Participants were only interviewed on topics for which they were above the median. Recruitment stopped when theme saturation was reached. Participants received a $50 gift card for each interview.

### Interview guide

The interview guide was developed based on literature review and Choosing Wisely recommendations [4]. Questions assessed knowledge of the Choosing Wisely Campaign and sought to identify drivers of overuse. We developed separate interviews for each topic. Interviews began by asking physicians how they would approach clinical scenarios that address the topics of interest, then to explain the thought process behind their actions. Open-ended questions were followed by discrete questions asking for levels of agreement with specific statements using Likert-type 5-point scales (e.g., “Patients who have been screened or prostate cancer expect to continue screening when they are older”). This method was used to more completely identify participants’ attitudes, beliefs and opinions.

### Data collection and analysis

Two investigators (T.A.R. and T.B.) conducted the 19 interviews in person or by phone. One investigator is board certified in internal medicine and geriatric medicine. The other is a senior research manager with over 12 years of experience conducting research in primary care. Interviews were transcribed verbatim and analyzed. The transcripts were reviewed after each interview and assessed for the emergence of new ideas or themes. We conducted additional interviews until no new ideas emerged and theme saturation was reached. Standard techniques of directed qualitative content analysis were used to code the transcripts [12, 13]. A preliminary coding scheme based on the interview guide was iteratively refined and applied to analyze the data using the constant comparative approach. The two investigators independently coded all transcripts. Discrepancies were resolved through discussion; consensus was reached among coders. Major themes were generated using constant comparative methods. Descriptive statistics including the mean and median were used to analyze the Likert-type scale responses.

## Results

Eighty physicians above the median were emailed to participate. We interviewed initial respondents and then sent additional recruitment emails to non-responders and stopped recruiting when interviews stopped generating new themes. Fourteen physicians who were above the median for overuse (8 women, 6 men) participated in nineteen topic-specific interviews. Physicians all practiced primary care and were board-certified in either family practice, internal medicine or geriatrics. We identified 4 major themes (Table 1). These are presented herein and illustrated using representative quotes (Table 2).

**Table 1:** Major themes and Subthemes Summarizing Clinician Views on Guidelines in Older adults

**Theme 1: Clinicians Have Heard About Guidelines for Older Adults**
Subthemes:
*Awareness that Guidelines Exist*
*Knowledge of guidelines*
**Theme 2: Reliance on Patient Preference**
**Theme 3: Improper Weighting of Potential Benefits and Harms**
Subthemes:
*Overestimation of benefits*
Underestimation of harms
**Theme 4: Resistance to change**

**Table 2:** Represenative quotes

Themes:	Examples
Awareness of Guidelines	“I would actively dissuade them from proceeding…I would tell them about possible down sides to going down the whole road…” [PSA] “I am totally satisfied with a hga1c in the 7–7.5 range. I don't there is any evidence of benefit below that.” [DM]
Reliance on Patient Preference	“Patient is seriously interested in it. Recognizes the downsides and false alarms…Patient preference matter a lot to me.” [PSA] “Patient preference or reassurance. A lot of that is nuance and depends on the patient and how much they know about their condition.” [UA/UC]
Clinical Uncertainty	Would be concerned they would get septic or sick. She is old [UA/UC] “Want to prevent escalation of infection…” [UA/UC] “Part of it depends on different types of 80. Some are more like 60 year olds” [DM]
Resistance to Change	“If this patient is doing well, I might leave it alone.” [DM] “For a 77 otherwise in good health, generally I would do test….But generally I won’t have a long discussion in terms of whether they should do it or not.” [PSA] “After long discussion, seems overwhelming majority of patients want to do this test. Given limits of time we have, I’ve been less detailed in discussion…” [PSA]

### Theme 1: Recognition of Guidelines

#### Awareness that guidelines exist

Even though participants were recruited from physicians above the median for overuse of the topic in question, participants voiced awareness of clinical recommendations to avoid overuse in older adults. When asking how one might approach PSA testing in an older man, one physician commented: “I'd just tell him the guidelines and see what his response was.” When asked how one would approach urinary testing in an older woman without specific genitourinary signs and symptoms, another participant responded: “we know now not to test women for a urinary tract infection without symptoms.”

#### Knowledge of guideline content

Participants were aware of guideline content, suggesting over testing was not due to a knowledge deficit. This was especially evident for diabetes. One physician commented: “We’ve always known to be a little more lenient for elderly patients because of their risk of hypoglycemia.” No participants referenced a specific guideline, but several recognized how guidelines have changed over the past few years: “I believe that the new guidelines state that a HbA1C up to 8 is acceptable.”

General knowledge of guideline content was also apparent in the responses to discrete questions. For example, 67% of physician responses were in agreement with a statement that harms of prostate cancer screening outweighed benefits for the average 77-year old man.

### Theme 2: Reliance on Patient Preference

Though participants were aware and knowledgeable of guidelines, many deferred to patient preference when deciding whether to order a test. One commented that it depended on: “whether or not the patient wants it done,” and would not necessarily try to dissuade the patient from testing. Several participants suggested they would continue testing or treatment because it was what patients expect or that it would make them less anxious: “If I couldn’t educate him enough to reason with him then I think I would order it to make sure the patient is less anxious.” Additionally, many participants stated they would order a test if the patient wanted the test done, even though they felt it was not indicated. Regarding PSA one physician commented: “If patient is very adamant about doing it, otherwise I usually don’t do it”. Another participant recognized that PSA screening in older men probably should not be ordered, but stated: “some patients are very insistent or expectant that they want to continue to get a PSA.”

### Theme 3: Improper Weighting of Potential Benefits and Harms

Almost all participants discussed their rationale for ordering or not ordering a test based on the perceived benefits, but few discussed harms associated with testing or treatment.

#### Overestimation of benefits

Participants seemed to overestimate benefits of testing. This was most apparent when rationalizing urinary testing in women with non-specific signs or symptoms. When discussing why they would order a urine study one participant discussed their: “concern for missing something that would turn into sepsis” and another mentioned they would: “want to prevent escalation of infection.” Results from the discreet questions revealed that half of participants were concerned they would miss a urinary tract infection that could become severe if they did not order urine studies in an older woman with non-specific symptoms such as fatigue.

#### Underestimation of harms

Few participants specifically discussed downsides of testing or treatment with patients. None mentioned that overusing urine testing could lead to overuse of antibiotics and few participants discussed the downsides of screening with PSA testing. One participant mentioned they would discuss potential harms of PSA screening: “I would tell them about possible down sides to going down that whole road-complications from biopsies, the invasive nature of it, knee jerk reaction to treat and do invasive things that might have side effects from treatment or diagnosis”, but later discussed not wanting to miss prostate cancer in an older healthy man. Participants did tend to recognize harms associated with overtesting when asked specifically about them in the discreet questions. For example, most (75%) participants agreed that harms associated with a HbA1C < 7.0 outweighed benefits in the average 77 year- old treated with insulin or a sulfonylurea.

### Theme 4: Resistance to Change

Many participants discussed the desire to not change management for patients doing well, preferring to maintain the status quo. For example, when discussing diabetes, one physician mentioned: “I wouldn’t shoot for a HbA1C < 6.7, but I wouldn’t necessarily change it”. Time constraints seemed to influence resistance to change: “After long discussion, seems overwhelming majority of patients want to do this test. Given limits of time we have, I’ve been less detailed in discussion.”

## Discussion

We characterized primary care physicians’ perspectives for overusing tests and treatments that are not indicated in older adults, targeting physicians who were frequent high users of: PSA screening in men aged ≥ 76 years, urine testing in women aged ≥ 65 years without specific genitourinary or infectious signs or symptoms, and prescribing insulin or oral hypoglycemic medication in adults aged ≥ 75 years with diabetes and HbA1C < 7.0%. Although participants were selected for prior overuse, most were generally aware of guidelines and their recommendations. Knowledge deficits often are not the most significant driver of guideline non-adherence, and our data support this [11, 14]. Even among clinicians who deviate from guidelines frequently we find that they have an understanding of guidelines.

Deference to perceived patient preferences often influenced the decision to order testing and treatment, especially for PSA screening. Recent qualitative data suggests that in fact, many patients would be amendable to stopping cancer screening, but few recall actually discussing screening with a clinician [15]. We also found that physicians often assume a patient’s preference is to continue to be screened for prostate cancer if they had been screened in the past. Thus, physicians may not be communicating the risks of additional testing or treatment because they incorrectly assume patients want to continue their care as is. Physicians who over order may be making more assumptions about patient preferences or be less prone to challenge patient views.

Overestimation of benefits and underestimation of harms was a central theme throughout all three topics. For urinary testing, most clinicians expressed concern about missing a severe urinary tract infection without highlighting risks associated with urine testing such as overuse of antibiotics. Several studies have shown that ordering urine testing in asymptomatic women leads to unnecessary antibiotics [7, 16] and that PSA screening in asymptomatic older men can lead to negative consequences such as harms from biopsies, surgery or radiation treatment, as well as psychological harm [17]. There is also literature to support that clinicians want to feel like they have done everything for their patients [18]. Participating physicians seemed to under-weigh the risks of performing the action when discussing how they approach patient care, though acknowledged these risks when specifically asked general knowledge questions. Other studies have suggested that though clinicians have knowledge of clinical guidelines, they may have inadequate knowledge about harms of cancer screening [9, 15]. Thus, when approaching shared decision-making, they may underestimate and not fully communicate potential harms of some actions.

Finally, reluctance to change if a patient is doing well was often mentioned when asked about diabetes. Again, clinicians did not seem to recognize the harms associated with overtreatment citing the fact that if the patient was doing fine, they would not make adjustments, specifically because doing this would take time. This compares to literature citing concerns with inefficiency and inertia of previous practice [18, 19].

These data suggest that interventions to reduce overuse should not merely convey clinical recommendations but rather should target the psychological motivations that lead to overuse. Decision support tools that increase clinicians’ attention to potential downsides of an easily ordered test or a patient’s current treatment may improve care more than simple clinical guidance alone. Additionally, fostering clinician communication skills about potential harms may help reduce overutilization, especially for prostate cancer screening [20].

There are several limitations to this study. First, we included only primary care physicians in one health system, so our results may not be generalizable to other systems or types of clinicians. Second, we choose three specific overuse topics. It is unknown if results would be similar for others. Third, we did not compare differences between physicians with high and low levels of over ordering. Thus, it is unclear if there these groups have different attitudes towards overuse or if they have similar views but are applied differently. Fourth, participant variables such as years in practice or medical school graduation date were not collected. Finally, observations from physicians willing to participate in this study may not be generalizable to other physicians.

## Conclusion

We found several reasons why primary care physicians may not follow guidelines to limit overuse of testing and treatment in older adults. Approaches to decrease unnecessary testing and treatment in older adults should account for perceived patient preferences, make the realistic harms of overtesting and overtreatment salient, and address clinical inertia among patients who are over treated but appear to be doing well.

## Supplementary Information

**Additional file 1 **

**Additional file 2 **

**Additional file 3 **

## Data Availability

Additional data is available upon request.

## Abbreviations

PSA: Prostate Specific Antigen
HbA1C: Hemoglobin A1C
