# Supplementary material for: Examining primary care physician rationale for not following geriatric choosing wisely recommendations
Source: BMC Fam Pract. 2021 May 15;22:95. doi: 10.1186/s12875-021-01440-w (PMC8126116; doi:10.1186/s12875-021-01440-w)
Supplement: Supplementary file 1 — Additional file 1 [file 12875_2021_1440_MOESM1_ESM.pdf]

DM interview

Record ID ______________________

Study ID (from recruitment file) ______________________

Date of Interview ______________________

Interviewer Name ______________________

Is this interview being audio-recorded?
- ◯ Yes
- ◯ No

Hello, my name is X and I am conducting a study funded by the National Institutes of Health that is examining clinical decision making for older adults. I would like to interview you about how clinicians make decisions about hemoglobin A1c goals for older diabetes patients. Imagine that you are seeing an 80 year woman for a diabetes follow up visit. She uses glargine insulin 45 units once daily and metformin 1000 mg twice daily for her diabetes. The hemoglobin A1c is 6.7%. She is not having symptoms of hypoglycemia. When she checks her morning glucose she gets readings between 85 and 120 mg/dl.

Please describe your approach to selecting a HbA1c treatment goal for a patient like this. ______________________

What do you do when the HbA1c is lower than your goal? Do you have an approach to de-escalating or stopping diabetic medications in older adults? Can you describe situations where you stopped or reduced a medication for diabetes? ______________________

Explain what the most important medical or non-medical factors that influence when you decide to reduce diabetes treatment? Are there specific conditions or comorbidities you use to make your decision? ______________________

Has your approach changed over the past few years? ______________________

For each of the following statements, I would like you to tell me if you: strongly agree, agree, neither agree nor disagree, disagree, strongly disagree.

Patients who have been told to keep their diabetes tightly controlled in the past believe when they need to maintain the same level of control when they are older.
- ◯ Strongly agree
- ◯ Agree
- ◯ Neither agree nor disagree
- ◯ Disagree
- ◯ Strongly disagree

The potential health benefits of keeping the HbA1c below 7.5 outweigh the potential harms for the average 77 year old insulin or sulfonylurea treated diabetes patient.

- ◯ Strongly agree
- ◯ Agree
- ◯ Neither agree nor disagree
- ◯ Disagree
- ◯ Strongly disagree

The potential health benefits of keeping the HbA1c below 7.0 outweigh the potential harms for the average 77 year old insulin or sulfonylurea treated diabetes patient.

- ◯ Strongly agree
- ◯ Agree
- ◯ Neither agree nor disagree
- ◯ Disagree
- ◯ Strongly disagree

The potential health benefits of keeping the HbA1c below 7.0 outweigh the potential harms for the average 85 year old insulin or sulfonylurea treated diabetes patient.

- ◯ Strongly agree
- ◯ Agree
- ◯ Neither agree nor disagree
- ◯ Disagree
- ◯ Strongly disagree

I would be concerned that if I told an older diabetes patient that I recommended letting the HbA1c go higher, he or she would be offended.

- ◯ Strongly agree
- ◯ Agree
- ◯ Neither agree nor disagree
- ◯ Disagree
- ◯ Strongly disagree

For insulin or sulfonylurea-treated patients over 75, I am usually able to tell when someone is at risk from hypoglycemia by asking about symptoms.

- ◯ Strongly agree
- ◯ Agree
- ◯ Neither agree nor disagree
- ◯ Disagree
- ◯ Strongly disagree

Most of my primary care colleagues would not reduce insulin or sulfonylurea dose for elderly patients with a HbA1c < 7 unless there were symptoms of hypoglycemia

- ◯ Strongly agree
- ◯ Agree
- ◯ Neither agree nor disagree
- ◯ Disagree
- ◯ Strongly disagree

In my experience, I have not found symptomatic hypoglycemia to be a big problem for my older insulin or sulfonylurea treated patients with diabetes.

- ◯ Strongly agree
- ◯ Agree
- ◯ Neither agree nor disagree
- ◯ Disagree
- ◯ Strongly disagree

Thank you! That is the end of the interview. We appreciate your participation. We'd like to mail you a $50 Target gift card. What is best address to mail this to?

____

Interviewer notes

____
