# Supplementary material for: Examining primary care physician rationale for not following geriatric choosing wisely recommendations
Source: BMC Fam Pract. 2021 May 15;22:95. doi: 10.1186/s12875-021-01440-w (PMC8126116; doi:10.1186/s12875-021-01440-w)
Supplement: Supplementary file 2 — Additional file 2 [file 12875_2021_1440_MOESM2_ESM.pdf]

PSA interview

Record ID ______________________

Study ID (from recruitment file) ______________________

Date of interview ______________________

Interviewer name ______________________

Is the interview being audio-recorded?
- ◯ Yes
- ◯ No

Hello, my name is X and I am conducting a study funded by the National Institutes of Health that is examining clinical decision making for older adults. I would like to interview you about how clinicians make decisions about using the PSA test in older men who do not have a history of prostate cancer.

Imagine that you are seeing a 77 year old man for an annual visit during a typical work day. He received a screening PSA test at his last physical.

Please describe how you would typically approach the PSA test for this individual. ______________________

Explain what the most important medical or non-medical factors are that influence how you would proceed. ______________________

When you do order a screening PSA test in cases like this (if any), what are the most important reasons why you order it? ______________________

When you do not order a screening PSA test in cases like this (if any), what are the most important reasons why you do not order it? ______________________

For each of the following statements, I would like you to tell me if you: strongly agree, agree, neither agree nor disagree, disagree, strongly disagree.

Patients who have been getting screened for prostate cancer in the past expect to continue screening when they are older.
- ◯ Strongly agree
- ◯ Agree
- ◯ Neither agree nor disagree
- ◯ Disagree
- ◯ Strongly disagree

The potential health benefits of doing a PSA test outweigh the potential harms for the average 77 year old man.
- ◯ Strongly agree
- ◯ Agree
- ◯ Neither agree nor disagree
- ◯ Disagree
- ◯ Strongly disagree

The potential health benefits of doing a PSA test outweigh the potential harms for the average 82 year old man.

◯ Strongly agree
◯ Agree
◯ Neither agree nor disagree
◯ Disagree
◯ Strongly disagree

Discussing when to stop prostate cancer screening with a patient takes a lot of time.

◯ Strongly agree
◯ Agree
◯ Neither agree nor disagree
◯ Disagree
◯ Strongly disagree

I would be concerned that if I told an older man that I no longer recommended PSA screening above a certain age, he would be offended.

◯ Strongly agree
◯ Agree
◯ Neither agree nor disagree
◯ Disagree
◯ Strongly disagree

For men over 75, I am more concerned about failing to prevent someone from getting metastatic prostate cancer than I am about the potential harms associated with diagnosing and treating early prostate cancer.

◯ Strongly agree
◯ Agree
◯ Neither agree nor disagree
◯ Disagree
◯ Strongly disagree

Most urologists routinely check PSA levels in men with no history of prostate cancer who are over 75 years old.

◯ Strongly agree
◯ Agree
◯ Neither agree nor disagree
◯ Disagree
◯ Strongly disagree

Most of my primary care colleagues routinely check PSA levels in men with no history of prostate cancer who are over 75 years old.

◯ Strongly agree
◯ Agree
◯ Neither agree nor disagree
◯ Disagree
◯ Strongly disagree

For my patients (like one I mentioned) who have been getting regular screening PSA tests, I would order the test again without discussing with them.

◯ Strongly agree
◯ Agree
◯ Neither agree nor disagree
◯ Disagree
◯ Strongly disagree

Please tell me the top 3 most important reasons you would use a PSA screening test in someone over 75.

____

Please tell me the 3 most important reasons for not doing a PSA screening test in someone over 75.

____

Thank you! That is the end of the interview. We appreciate your participation. We'd like to mail you a $50 Target gift card. What is best address to mail this to?

____

Interviewer notes

____
