# Supplementary material for: Examining primary care physician rationale for not following geriatric choosing wisely recommendations
Source: BMC Fam Pract. 2021 May 15;22:95. doi: 10.1186/s12875-021-01440-w (PMC8126116; doi:10.1186/s12875-021-01440-w)
Supplement: Supplementary file 3 — Additional file 3 [file 12875_2021_1440_MOESM3_ESM.pdf]

UA interview

Record ID ________________

Study ID (from recruitment file) ________________

Date of Interview ________________

Interviewer name ________________

Is this interview being audio-recorded?
◯ Yes
◯ No

Hello, my name is X and I am conducting a study funded by the National Institutes of Health that is examining clinical decision making for older adults. I would like to interview you about how and when clinicians order urine studies (e.g., urinalysis and urine culture) in older women aged ≥ 65 years.

Scenario A

Imagine that you are seeing an 84-year-old woman for a visit during a typical work day. She is coming in for chronic urinary frequency and incontinence. She has urinary frequency (every 2-3 hours) and nocturia (every 3 hours). During the past few months, she thinks she has noticed an increase in frequency and incontinence and started wearing pads during the day. She has no dysuria or hematuria. No fevers. She has tried medications for over active bladder (oxybutynin, tolterodine) in the past that were not very effective. She's had multiple urine cultures for urinary incontinence and has been treated with antibiotics for positive urine cultures approximately 3 times in the past year. She has a diagnosis of recurrent UTIs listed in her past medical history.

Please describe how you would typically approach urinary testing in the patient. ________________

Explain what the most important medical or non-medical factors are that influence how you would proceed with urine testing. ________________

When you do order urine studies (e.g., urinalysis and/or urine culture) in cases like this, what are the most important reasons why you order them? ________________

When you do not order urine studies in cases like this, what are the most important reasons why you do not order them? ________________

For each of the following statements, I would like you to tell me if you: strongly agree, agree, neither agree nor disagree, disagree, strongly disagree.

Patients who have been worked up for UTI with a urinalysis and/or urine culture for chronic incontinence and frequency in the past expect to continue to be tested for UTI with any changes in frequency and incontinence?

- ◯ Strongly Agree
- ◯ Agree
- ◯ Neither agree nor disagree
- ◯ Disagree
- ◯ Strongly disagree

The potential benefits of doing urine studies outweigh the potential harms for older women with chronic urinary symptoms.

- ◯ Strongly agree
- ◯ Agree
- ◯ Neither agree nor disagree
- ◯ Disagree
- ◯ Strongly disagree

Discussing with the patient why you are not ordering urine studies right away takes a lot of time.

- ◯ Strongly agree
- ◯ Agree
- ◯ Neither agree nor disagree
- ◯ Disagree
- ◯ Strongly disagree

I would feel comfortable not ordering urine studies for a patient like this, and asking her to come back if her symptoms worsened or she developed new symptoms such as dysuria, fever or hematuria.

- ◯ Strongly agree
- ◯ Agree
- ◯ Neither agree nor disagree
- ◯ Disagree
- ◯ Strongly disagree

I would be concerned that if I did not order urine studies during this visit, I would miss a urinary tract infection that may evolve into pyelonephritis and/or urosepsis.

- ◯ Strongly agree
- ◯ Agree
- ◯ Neither agree nor disagree
- ◯ Disagree
- ◯ Strongly disagree

Most of my colleagues (e.g., other internists or geriatricians) would order urine studies for this patient.

- ◯ Strongly agree
- ◯ Agree
- ◯ Neither agree nor disagree
- ◯ Disagree
- ◯ Strongly disagree

For my patients (like the one I mentioned), I would order urine testing for UTI without discussing the benefits or risks with my patient.

- ◯ Strongly agree
- ◯ Agree
- ◯ Neither agree nor disagree
- ◯ Disagree
- ◯ Strongly disagree

For my patients (like the one I mentioned) I would order antibiotics for suspected UTI in addition to urine studies.

- ◯ Strongly agree
- ◯ Agree
- ◯ Neither agree nor disagree
- ◯ Disagree
- ◯ Strongly disagree

Please tell me the top 3 most important reasons you would order a urinalysis and/or urine culture during this visit.

____

Please tell me the 3 most important reasons for not ordering a urinalysis and/or urine culture during this visit.

____

Scenario B
Imagine that you are seeing an 84-year-old woman with cognitive impairment for a visit during a typical work day. She is accompanied by her daughter. She is coming in with worsening confusion and fatigue. She has also been eating and drinking less. She's had multiple urine cultures ordered in her chart and has been treated with antibiotics for positive urine cultures approximately 3 times in the past year. She has a diagnosis of recurrent UTIs listed in her past medical history. She has no fevers, or reported chills. Her daughter requests work-up for a urinary tract infection because this always happens when she gets a urine infection.

Please describe how you would typically approach urinary testing in the patient. ______________________________

Explain what the most important medical or non-medical factors are that influence how you would proceed with urine testing. ______________________________

When you do order urine studies (e.g., urinalysis and/or urine culture) in cases like this, what are the most important reasons why you order them? ______________________________

When you do not order urine studies in cases like this, what are the most important reasons why you do not order them? ______________________________

For each of the following statements, I would like you to tell me if you: strongly agree, agree, neither agree nor disagree, disagree, strongly disagree.

Patients (and/or their caregivers) who have been worked up for UTI with a urinalysis and/or urine culture for non-specific symptoms (or non-genitourinary signs or symptoms) in the past expect to continue to be tested for UTI when they develop non-specific symptoms.

- ◯ Strongly agree
- ◯ Agree
- ◯ Neither agree nor disagree
- ◯ Disagree
- ◯ Strongly disagree

The potential benefits of doing urine studies outweigh the potential harms for older women with cognitive impairment who have only non-specific symptoms (no urinary tract symptoms).

- ◯ Strongly agree
- ◯ Agree
- ◯ Neither agree nor disagree
- ◯ Disagree
- ◯ Strongly disagree

Discussing with the patient and her family why you are not ordering urine studies right away takes a lot of time.

- ◯ Strongly agree
- ◯ Agree
- ◯ Neither agree nor disagree
- ◯ Disagree
- ◯ Strongly disagree

For a patient like this one, I would feel comfortable not ordering urine studies, and asking the patient to come back if her symptoms worsened or she developed new symptoms such as dysuria, fever or hematuria.

- ◯ Strongly agree
- ◯ Agree
- ◯ Neither agree nor disagree
- ◯ Disagree
- ◯ Strongly disagree

I would be concerned that if I did not order urine studies during a visit for a patient like this, I would miss a urinary tract infection that may evolve into pyelonephritis and/or urosepsis.

- ◯ Strongly agree
- ◯ Agree
- ◯ Neither agree nor disagree
- ◯ Disagree
- ◯ Strongly disagree

Most of my colleagues (e.g., other internists or geriatricians) would order urine studies for this patient.

- ◯ Strongly agree
- ◯ Agree
- ◯ Neither agree nor disagree
- ◯ Disagree
- ◯ Strongly disagree

For my patients (like the one I mentioned), I would order urine testing for UTI without discussing the benefits or risks with my patient.

- ◯ Strongly agree
- ◯ Agree
- ◯ Neither agree nor disagree
- ◯ Disagree
- ◯ Strongly disagree

For my patients (like the one I mentioned) I would order antibiotics for suspected UTI in addition to urine studies.

- ◯ Strongly agree
- ◯ Agree
- ◯ Neither agree nor disagree
- ◯ Disagree
- ◯ Strongly disagree

Please tell me the top 3 most important reasons you would order a urinalysis and/or urine culture during this visit for a patient like this.

____

Please tell me the 3 most important reasons for not ordering a urinalysis and/or urine culture during this visit.

____

Thank you! That is the end of the interview. We appreciate your participation. We'd like to mail you a $50 Target gift card. What is best address to mail this to?

____

Interviewer notes

____
